# Supplementary material for: Impact of a virtual learning environment on the conscious prescription of antibiotics among Colombian dentists
Source: PLoS One. 2022 Jan 28;17(1):e0262731. doi: 10.1371/journal.pone.0262731 (PMC8797226; doi:10.1371/journal.pone.0262731)
Supplement: S1 Appendix — Impact on the number of participants who correctly answered questions from the questionnaire. (DOCX) [file pone.0262731.s001.docx]

Supporting information

**S1 Appendix. Immediate and 6-month impact of the virtual learning environment (VLE) implementation.** Impact on the number of participants who correctly answered questions from the questionnaire

| Correct answer | Before  n (%) | Immediate  n (%) | p value | Before  n (%) | 6-months | p value |
| --- | --- | --- | --- | --- | --- | --- |
| Awareness | | | | | | |
| Useful on bacteria | 206(100) | 206(100) | 1.000 | 155 (100) | 155 (100) | 1.000 |
| Non useful on viruses | 183 (88.8) | 202 (97.6) | 0.000*** | 136 (87.7) | 150 (96.8) | 0.001** |
| Non useful on fungi | 167 (81.1) | 193 (93.2) | 0.000*** | 124 (80) | 138 (89.0) | 0.024* |
| Definition of antibiotic resistance | 149 (72.3) | 170 (82.1) | 0.013* | 111 (71.6) | 98 (63.2) | 0.092 |
| Frequent use of antibiotics reduces efficacy | 168 (81.6) | 188 (90.8) | 0.002** | 123 (79.4) | 138 (89.0) | 0.014* |
| Antibiotic prescription contributes to antibiotic resistance | 101 (49) | 157 (75.8) | 0.000*** | 73 (47.1) | 96 (61.9) | 0.006** |
| Attitudes | | | | | | |
| Prescription based on patient’s symptoms | 84 (40.8) | 107 (51.7) | 0.017* | 71 (45.8) | 79 (51.0) | 0.322 |
| No prescription following patient´s preferences | 188 (91.3) | 189 (91.3) | 1.000 | 142 (91.6) | 139 (89.7) | 0.648 |
| Prescription based on knowledge acquired in undergraduate and graduate courses | 151 (73.3) | 153 (73.9) | 0.892 | 115 (74.2) | 126 (81.3) | 0.117 |
| No prescription based on antibiotics availability at the nearest pharmacy | 183 (88.8) | 185 (89.8) | 0.851 | 139 (89.7) | 139 (89.7) | 1.000 |
| Prescription based on recommendations by expert colleagues | 155 (75.2) | 155 (74.9) | 1.000 | 118 (76.1) | 43 (27.7) | 0.000*** |
| No prescription when under pressure by the time | 191 (92.7) | 200 (97.1) | 0.064 | 144 (92.9) | 150 (96.8) | 0.522 |
| No prescription when under pressure or request by patient´s companion | 199 (96.6) | 202 (98.1) | 0.549 | 147 (94.8) | 148 (95.5) | 0.956 |
| Prescription based on international guidelines | 38 (18.4) | 88 (42.5) | 0.000*** | 31 (20) | 50 (32.3) | 0.014* |
| Intention to practice | | | | | | |
| Antibiotic prescription during daily dental practice is uncommon | 54 (26.2) | 94 (45.4) | 0.000*** | 40 (25.8) | 60 (38.7) | 0.004** |
| No antibiotic prescription for non-surgical periodontal treatment | 190 (92.2) | 199 (96.1) | 0.096 | 145 (93.5) | 148 (95.5) | 0.508 |
| No antibiotic prescription for acute gingivitis and stomatitis | 180 (87.4) | 193 (93.2) | 0.036* | 136 (87.7) | 139 (89.7) | 0.648 |
| No antibiotics prescription for pain treatment | 198 (96.1) | 203 (98.1) | 0.289 | 149 (96.1) | 155 (100) | -- |
| No antibiotic prescription when an anesthetic complication is presented | 200 (97.6) | 203 (98.1) | 1.000 | 150 (97.4) | 151 (97.4) | 1.000 |
| No antibiotic prescription after simple tooth extraction | 171 (41.4) | 201 (97.1) | 0.000*** | 132 (85.2) | 147 (94.8) | 0.001** |
| No antibiotic prescription after endodontic treatment | 178 (86.4) | 199 (96.6) | 0.000*** | 137 (88.4) | 141 (91.0) | 0.503 |
| No antibiotic prescription after dental implant placement | 129 (62.6) | 172 (83.5) | 0.000*** | 100 (64.5) | 112 (72.3) | 0.088 |
| Antibiotic prescription for abscesses of dental origin with fever | 191 (92.7) | 197 (95.6) | 0.332 | 143 (92.3) | 145 (93.5) | 0.733 |
| Antibiotic prescription on a patient at increased risk of developing IE | 181 (87.9) | 185 (89.8) | 0.701 | 133 (85.8) | 137 (88.4) | 0.682 |
| Number of days for a first-choice antibiotic prescription | 186 (90.3) | 193 (93.2) | 0.238 | 152 (91.6) | 143 (92.3) | 1.000 |
| No antibiotic prophylaxis for patients with diabetes mellitus | 158 (76.7) | 158 (76.3) | 1.000 | 127 (81.9) | 124 (80.0) | 0.711 |
| No antibiotic prophylaxis for patients with autoimmune diseases | 154 (74.8) | 129 (62.3) | 0.005** | 119 (76.8) | 126 (81.3) | 0.360 |
| No antibiotic prophylaxis for patients under immunosuppressive therapy | 138 (67.0) | 120 (58.0) | 0.037* | 107 (69.0) | 119 (76.8) | 0.104 |
| No antibiotic prophylaxis for patients with AIDS | 145 (70.4) | 123 (59.4) | 0.017* | 115 (74.2) | 127 (81.9) | 0.096 |
| Antibiotic prophylaxis for patients with rheumatic heart disease | 137 (66.5) | 108 (52.2) | 0.001** | 100 (64.5) | 97 (62.6) | 0.775 |
| Antibiotic prophylaxis for patients with coronary bypass surgery | 135 (65.5) | 109 (52.7) | 0.006** | 101 (65.2) | 97 (62.6) | 0.683 |
| No antibiotic prophylaxis for patients with pacemakers | 84 (40.8) | 117 (56.5) | 0.000*** | 87 (56.1) | 97 (62.6) | 0.009** |
| Antibiotic prophylaxis for patients with IE | 173 (84.0) | 180 (87.0) | 0.451 | 124 (80) | 131 (84.5) | 0.281 |
| Antibiotic prophylaxis for patients with cardiac valve prosthetics | 169 (82.0) | 171 (82.6) | 1.000 | 128 (82.6) | 122 (78.7) | 0.430 |
| Antibiotic prophylaxis for patients with joint prosthetics | 135 (65.5) | 136 (65.7) | 1.000 | 108 (69.7) | 110 (71.0) | 0.896 |
| Antibiotic prophylaxis for patients with ventricular septum defect | 114 (55.3) | 99 (47.8) | 0.133 | 81 (52.3) | 85 (54.8) | 0.708 |

*p < 0.05, **p < 0.01, ***p < 0.001
