# Supplementary material for: Impact of a virtual learning environment on the conscious prescription of antibiotics among Colombian dentists
Source: PLoS One. 2022 Jan 28;17(1):e0262731. doi: 10.1371/journal.pone.0262731 (PMC8797226; doi:10.1371/journal.pone.0262731)
Supplement: Attachment — Submitted filename: Responses reviewers F.docx [file pone.0262731.s002.docx]

Dear Editor:

Prof. Dr. Andrej M Kielbassa

Academic Editor

PLOS ONE

Please, in the attached file, you will find the revised manuscript PONE-D-21-04062: " Impact of a Virtual Learning Environment on the conscious prescription of antibiotics in Colombian dentists" by Angarita Diaz *et al.*, that we are submitting for publication in "PLOS ONE". We have added responses to the suggestions and comments of the reviewers and would like to thank them for all their time and consideration of our work.

Best regards,

The authors

***RESPONSE JOURNAL REQUIREMENTS:***

-Comments to the Author

-Please ensure that your manuscript meets PLOS ONE's style requirements, including those for file naming.

**Response:** We confirm that our manuscript meets PLOS ONE’s style requirements. Also, the file naming meets the requested style.

-We note that Figure 1 in your submission contain copyrighted images. All PLOS content is published under the Creative Commons Attribution License (CC BY 4.0), which means that the manuscript, images, and Supporting Information files will be freely available online, and any third party is permitted to access, download, copy, distribute, and use these materials in any way, even commercially, with proper attribution.

**Response:** The images used in this manuscript contain the Creative Commons Attribution License (CC BY 4.0). The course is a product of this research and is Open Access.

We uploaded the completed Content Permission Form in our submission, and we added in the figure caption the following: Reprinted from [VLE “Prescripción consciente de Antibióticos en odontología”] under a CC BY license, with permission from [Universidad Cooperativa de Colombia], original copyright [2020]”. **Line 157 (manuscript with track changes).**

-We note that Figure 1 includes an image of a participant in the study.

**Response:** We inform you that Figure 1 does not include an image of a participant in the study. The person who appears is a teacher of the webinar. However, we reduced the size of this photography with the idea of avoiding the image of the person.

-Thank you for including your ethics statement on the online submission form:

"The present study was approved by the ethics subcommittee from Universidad Cooperativa de Colombia (No. 015-2018). A Written consent was obtained through the VLE.".

To help ensure that the wording of your manuscript is suitable for publication, would you please also add this statement at the beginning of the Methods section of your manuscript file (to include type of consent provided by participants).

**Response:** We added the request. **Line 137 (track changes).**

- Thank you for your response to our copyright queries. Could you please confirm whether the person in figure 1 is shown as part of the “Prescripción consciente de Antibióticos en odontología” under the CC BY 4.0 license? If this identifying image is being republished it will be acceptable to include their image, but otherwise it will need to be removed.

**Response:** We confirm that the person in figure 1 is part of the “Prescripción consciente de Antibióticos en odontología” under the CC BY 4.0 license.

***RESPONSE TO REVIEWERS***

***REVIEWER 1***

Dear reviewer, thanks for your comments that support us to improve the manuscript. We specify the changes and corrections made as follows:

-Comments to the Author

How were the participants included in each city? Was the sample probabilistic or non-probabilistic? It is necessary to describe more details.

**Response:** We included specifications about the sample and how we incorporated the participants in each city. **Line 143 (manuscript with track changes).**

- The authors must discuss the absence of a control group

**Response:** In the discussion we explained the absence of a control group. **Line 384 (track changes).**

- The objectives and the conclusions must be aligned; the authors did not cite the development of a Virtual Learning Environment in the objectives.

**Response:** We modified the objectives and conclusions, including the development of the Virtual Learning Environment in the objectives. **Line 131 and line 472 (track changes).**

- Why did dentists were unable to modify their attitudes on antibiotic prescription six months after the course, besides modifying their awareness? Due complexity of this theme, maybe is necessary a more profound discussion based on educational psychology. Courses cannot be the best strategy.

**Response:** In the discussion we explained why dentists may not be able to modify their attitudes on antibiotic prescription 6 months after the intervention. **Line 401 (track changes).**

***RESPONSE TO REVIEWERS***

***REVIEWER 2***

Dear reviewer, thanks for your comments that support us to improve the manuscript. We specify the changes and corrections made as follows:

Abstract

- Please see Guidelines for Authors,and consult some recently published Plos One papers. No subheadings mandatory here. Please delete "Background:", "Methods:", "Results:", and "Conclusion :". Revise carefully for proper spacebar use.

**Response:** We modified the abstract, deleting "Background", "Methods", "Results", and "Conclusion" and we revised spacebar use**. Line 30 (manuscript with track changes).**

- Please provide complete results and add exact P values on a 3-digit basis (example: p=0.109). Revise carefully.

**Response:** We added the p-value on a 3-digit basis throughout the manuscript. **Line 45 (track changes).**

- Remember that this section allows for 300 words, see Guidelines. Currently, your word count would be 216, and it seems unclear why you obviously have failed to provide a complete section presenting the most important outcome convincingly.

**Response:** We completed the abstract presenting the most important outcomes. **Line 30 (track changes).**

- With your conclusions, please do not simply repeat your results. Instead, stick to your aims, and provide a sound and reasonable extension of your outcome. This extension must be based on your results, but a simple repetition would not seem convincing.

**Response:** We modified the abstract without repeating the results. We sticked the conclusion to our aims. **Line 53 (track changes).**

- This section has been poorly elaborated only. There are 12 (!) (co-)authors of this draft, all of them having read and approved this manuscript, right? This should result in a flawless paper, without any typos, and with sound conclusions. I hope you will agree.

**Response:** Dear reviewer, thanks for your comments. We agree that the abstract needed a more thorough elaboration. We modified this section. **Line 30(track changes).**

Intro

- Would seem basically sound.

- Please provide your objectives. Remember that there is a clear difference between "aims" and "objectives".

**Response:**

We added the objectives. **Lin**e **131** **(track changes).**

- A sound null hypothesis is missing. Note that H0 must be deducible from the forgoing thoughts.

**Response:** We modified the last section of the introduction with the idea to make the hypothesis deductible. **Line 129 (track changes).**

Meths

- Do not use legal terms like "®", and so on. This is unusual with scientific writing, please delete.

**Response:** We deleted the legal terms. **Line 164 (track changes).**

- With ALL materials and methodologies (including statistical software), please use general names with your text, followed by (brand name; manufacturer, city, STATE (abbreviated, if US), country) in parentheses. Stick to semicolon. Revise thoroughly.

**Response:** We included brand name information, manufacturer, city, state, and country. **Line 164 and line 214 (track changes).**

Results

- Would seem sound.

Disc

- Stick to H0 when staring this section.

**Response:** We modified this section to improve the discussion of the hypothesis. **Line 367 and line 371 (track changes).**

- Do not use authors' names with your text. The latter will be acknowledged with your Reference list. Delete "Sikkens et al.", and focus on your main thoughts.

**Response:** We deleted authors' names. **Line 387 (track changes).**

- Same with "Soltanimehr et al", and with "Teoh et al.,"

**Response:** We deleted authors' names. **Line 394 and line 419 (track changes).**

- Please add some educated thoughts on the generalizability of your outcome, to Columbia, and to other countries.

**Response:** We included information about the generalization of our outcomes to Colombia and other Hispanic countries. **Line 453 (track changes).**

Conclusion

- Your aims were "(...) this study aimed to determine the impact of a VLE designed to improve awareness, attitudes and intention to practice on antibiotic prescription". With your conclusions, please stick exclusively to your aims, and provide a reasonable extension of your outcome.

**Response:** We wrote the conclusion having in mind the objective of the study. **Line 472 (track changes).**

- "High scores on course characteristics and applicability such as design, content, quality, organization, timing as well as in the acquisition of knowledge, acquisition of consciousness and intention to modify prescription were received." This might be right, but it does not stick to your aims. Revise carefully.

**Response:** In the conclusion, we deleted information about scores on course characteristics and applicability. **Line 472 (track changes).**

Refs

- Please see recent Plos One papers, and add pmid numbers.

**Response:** We added PMID numbers. **Line 484 (track changes).**

All in all, the authors have submitted a nice draft, considered interesting and easily intelligible. This paper should be worth following after major revisions.

***RESPONSE TO REVIEWERS***

***REVIEWER 3***

Dear reviewer, thanks for your comments that support us to improve the manuscript. We specify the changes and corrections made as follows:

-General - explaining some of the technical terminology would be helpful - I do not understand 'intention to practice' - does this mean intention to prescribe antibiotics within the US guidelines which you have highlighted as the basis for Colombian dentistry?

**Response:** In the introduction, we added information about the meaning of Awareness, Attitudes, and intention to practice. **Line 102**. In addition, we specified in the Methods section, subheading “Impact of the VLE on dentist”, “intention to practice” the following: according to the AHA and ADA guidelines, and recent studies **Line 194 (manuscript with track changes).**

-Introduction - Too many references - 37 in just 5 paragraphs. Please think really carefully about the best one or two to chose to make each point rather than including up to 5 citations per sentence. For example, your first sentence should include the FDI World Dental Federation's policy statement or white paper on tackling overprescribing of antibiotics in dentistry ahead of many of the other references. And in the opening sentence of your third paragraph, could I suggest a single systematic review which looked at why dentists prescribe antibiotics unnecessarily: https://academic.oup.com/jac/article/74/8/2139/5475276

**Response:** We reduced the references, including only the relevant studies. Moreover, we incorporated the FDI World Dental Federation's policy statement and the recommended systematic review. **Line 73, line 86, line 102, line 115, line 117, line 121 and line 526 (track changes)**.

-Introduction - para 2 - guidelines for appropriate dental antibiotic prescribing differ around the world so you need to be clear in this paper that you are referring to that which is deemed appropriate in Colombia. And I think you have a typo as periodontal abscesses are less likely to be life-threatening than periapical abscesses. Later in the same paragraph, you state that orthopaedic surgeons 'must' define the antibiotic regimen - this does not make sense given the first part of this sentence.

**Response:** We modified this paragraph according to the recommendations. **Line 79 and line 87** **(track changes).**

-Introduction - para 3 - other than the first sentence of this paragraph, much of it would sit more comfortably in the discussion section.

**Response:** We decided not to include this information because, in the methods section, we specified that the course was developed according to the results from our prior study and included the reference. **Line 151 (track changes).**

-Methods - Be clear about when you undertook this study - this is particularly important as you refer to it in the discussion section.

**Response:** In methods, we added the time frame of the study. **Line 141 (track changes).**

-Thinning out the detail to that which is essential will make this section much clearer. Don't repeat information which is already in the tables.

**Response:** We deleted information that is contained in the tables. **Line 167 (track changes).**

-And be careful of jargon: alpha risk and beta risk mean nothing to the general reader. Simply say that your sample size was calculated as 140 dentists based on a cut off for significance of p=0.05.

**Response:** We changed to “The sample size was calculated as 140 dentists based on a cut-off for significance of p=0.05”. **Line 143 (track changes).**

-Also, could you mention the date when the ethics committee approved it?

**Response:** We included the date when the ethics committee approved the study. **Line 137 (track changes).**

-Table 1 - this needs to be streamlined to get rid of superfluous detail (eg 'welcome message' and 'course detail')

**Response:** In table 1, we deleted non-relevant information. **Line 175 (track changes).**

-Results - Again don't repeat information from the tables in the text. For example in the second sentence just say 'Details of the sociodemographic characteristics of participating dentists are included in Table 2.' In spite of its title, you have included more than just sociodemographic characteristics in Table 2. Baseline levels of awareness, attitudes and 'intention to practice' should be in a separate table. And Table 3 has WAY too much detail for the paper. You need to find a way to summarise this into a format digestible by the reader - or put it into an on-line appendix.

**Response:** We deleted repeated information from the tables and separated the table containing baseline levels of awareness, attitudes, and intention to practice. In addition, we moved Table 3 as a supplemental appendix (S1 Appendix). **Line 229, line 235 and line 237 (track changes).**

-Discussion - the key messages from the paper need to come through this section much more clearly. Given the extensive use of the US guidelines in the rest of the paper, I was rather surprised that you didn't reference any of the US work on dental antibiotic stewardship from Suda, Durkin or Goff.

**Response:** We organized the ideas to present them more clearly. Also, we included references about US work on dental antibiotic stewardship from Suda, Durkin, and Goff. **Line 599, line 615 and line 439 (track changes)..**
