# Supplementary material for: Impact of a virtual learning environment on the conscious prescription of antibiotics among Colombian dentists
Source: PLoS One. 2022 Jan 28;17(1):e0262731. doi: 10.1371/journal.pone.0262731 (PMC8797226; doi:10.1371/journal.pone.0262731)
Supplement: Attachment — Submitted filename: Responses to reviewers.docx [file pone.0262731.s003.docx]

Dear Editor:

Prof. Dr. Andrej M Kielbassa

Academic Editor

PLOS ONE

Please, in the attached file, you will find the revised manuscript PONE-D-21-04062: " Impact of a Virtual Learning Environment on the conscious prescription of antibiotics in Colombian dentists" by Angarita Diaz *et al.*, that we are submitting for publication in "PLOS ONE". We have added responses to the suggestions and comments of the reviewers and would like to thank them for all their time and consideration of our work.

Best regards,

The authors

***RESPONSE JOURNAL REQUIREMENTS:***

**Reviewer #2:**

Dear reviewer, thanks for your comments that support us to improve the manuscript. We specify the changes and corrections made as follows:

Abstract

- Please note that "(p=0.000)" is not very probable. Please double check, and revise for "(p < 0.0001)". Please revise thoroughly throughout your text.

**Response:** We modified according to your suggestion, and to the guide: “P-values. Report exact p-values for all values greater than or equal to 0.001. P-values less than 0.001 may be expressed as p < 0.001”.

- Revise for Journal style. "(p=0.042)" must read "(p = 0.042)".

**Response:** We put the corresponding spaces.

- Double check for typos, see "(p=0,000)", and revise carefully. Please note that one flawless drafts will be considered ready to proceed, and please note that all 12 co-authors have approved this version, so such typos would not seem understandable.

**Response:** We corrected the typo mistakes.

Intro

- Still, English remains a concern, see "This number is expected to continue increasing (...)." Again, revise carefully.

**Response:** We modified “This number is expected to increase up to 10 million deaths per year by 2050, as anticipated by the British government”. **Line 57 (manuscript with track changes).** We recheck and improve the English of the entire document.

- Still, a sound null hypothesis is missing at the end of this section. Remember that H0 must be deducible from the foregoing thoughts.

**Response:** We added the null hypothesis: The null hypothesis of the study is that the development and implementation of a VLE on conscious antibiotic prescription in dentistry does not have a favorable impact on participants’ awareness, attitudes, and intention to practice. **Line 121 (manuscript with track changes)**

Meths

- Heading must read "Materials and methods". Even if boring, all co-authors are strongly encouraged to stick to <https://journals.plos.org/plosone/s/submission-guidelines>.

**Response:** We modified the mistake with the heading. **Line 126 (manuscript with track changes)**

- "The sample size was calculated as 140 dentists based on a cut-off for significance of p=0.05." This would seem hard to follow. Please provide your primary end-point for this cut-off value.

**Response:** We added information about the sample size: “The sample size was calculated for each city including dentists with a Dentistry degree until 2016 according to the last update from the Observatorio Laboral para la Educación through a paired sample t-test (repeated measures). By accepting an alpha risk of 0.05 (5%) and a beta risk of 0.1 (10% potential) in a bilateral contrast, a total of 140 dentists were required. A 15% loss to follow-up was estimated.” **Line 136 (manuscript with track changes)**

However, in this document, we specify that reviewer # 3 on may, wrote: Just say that your sample size was calculated as 140 dentists based on a significance cutoff of p = 0.05. *For that reason, we had deleted the information on the before copy.*

- Again, do not use legal terms with your text, delete "Corp".

**Response:** We deleted “Corp”. **Line 213 126 (manuscript with track changes).**

Results

- "(...) and baseline levels of awareness, attitudes, and intention to practice are shown in Table 3." Do not leave the reader alone with your Tables. Instead, you must guide the reader with your full text. Without doubling each and every result, please provide the most important outcomes.

**Response:** We included: “Most of the participants were female (69.9%), performed a private clinical practice (60.2%), and had more than 10 years of clinical experience (45.6%); dentists with or without a postgraduate formation were equal (50%) (Table 2). Furthermore, most dentists showed a medium level of awareness (57.3%) and attitudes (80.1%) and a high level of intention to practice (91.3%) (Table 3)” **Line 230 (manuscript with track changes).**

In this document, we specify that reviewer # 3 in the correction above, wrote: Again don't repeat information from the tables in the text. For example in the second sentence just say 'Details of the sociodemographic characteristics of participating dentists are included in Table 2.' *For that reason, we had deleted the information on the before copy.*

- Please double check and revise for correct parentheses, see "(before: 5.0 IQR [4.0-6.0], after 6.0, IQR [5.0-6.0], (p=0.000)", and compare to "(before: 5.0 IQR [4.0-5.25], after 5.0, IQR [4.0-6.0] p=0.000)". Revise carefully for uniform formatting.

**Response:** We compared and standardized the information.

- Regarding the p values, see comments given above, and revise carefully.

**Response:** We modified according to the guide and the reviewer.

Disc

- Refer to H0 when starting this section.

**Response:** We started the Discussion section with H0: “The findings from this study allowed us to reject the null hypothesis as significant differences in awareness and intention to practice of participants on antibiotic prescription were found”. **Line 372 (manuscript with track changes).**

Refs

- Again, please revise for uniform formatting. Again, it would seem astonishing why the 12 co-authors simply have ignored the previous recommendations (which would seem easy to follow).

**Response:** We modified according to the guide (which request doi) and recent Dentistry-Plos One papers (which request pmid).

- For example "Sofhauser C. Intention in nursing practice. nursing science quarterly. 2016;29(1):31-34. https://doi.org/10.1177/0894318415614629 PMID: 26660773." must read "Sofhauser C. Intention in nursing practice. Nurs Sci Q. 2016; 29(1): 31–34. https://doi.org/10.1177/0894318415614629 PMID: 26660773" Double check for spacebar use, correct Journal abbreviation, correct hyphen ("–", not "-"), and so on. Remember to re-submit a flawless manuscript, to avoid possible errors with the proofs. This is considered the Co-Authors' task, not the Reviewers' (or the Type-setter's) one.

In total, this revised and re-submitted draft has been considerably improved, but is not ready to proceed.

**Response:** We did check for spacebar use, corrected the journal abbreviation, and fixed the hyphen. Also, modified according to the guide, which request doi: “A DOI number for the full-text article is acceptable as an alternative to or in addition to traditional volume and page numbers. When providing a DOI, adhere to the format in the example above with both the label and full DOI included at the end of the reference (doi: 10.1016/j.molimm.2014.11.005). Do not provide a shortened DOI or the URL.”

**Reviewer #4:**

Dear reviewer, thanks for your comments that support us to improve the manuscript. We specify the changes and corrections made as follows:

This paper is about the prescription of antimicrobials in dentistry, and describes the implementation of a VLE course and it´s impacts on awareness, attitudes and intention to practice regarding antibiotic prescription in dentistry. In the face of the challenge of increasing microbial resistance, this is an important topic, and successful experiences must be reported. Thus, I think this article is worthy of publication. However, I have a few comments that might help to improve the quality of the article before publication. I will refer to the page and line numbers in the PDF version of the Revised Manuscript with Track Changes.

ABSTRACT:

- Line 45: the authors correctly followed the recommendation of reviewer 2 and modified the P values on a 3-digit basis. However, the notation when the value of p is less than 0.001 must be p<0.001, and not p=0.000. I suggest this modification throughout the article.

**Response:** We modified according to your suggestion and, to the guide: “P-values. Report exact p-values for all values greater than or equal to 0.001. P-values less than 0.001 may be expressed as p < 0.001”. **Line 42 (manuscript with track changes).**

INTRODUCTION:

- Line 129: although authors revised the text in response to reviewer 2's indication that a sound null hypothesis is missing, this H0 was not really clear. Perhaps a proposal is: "considering that the VLE is a teaching-learning method that may not improve awareness, attitudes, etc., we develop a VLE and measure the impact". I believe that the way of writing can make the null hypothesis clearer.

**Response:** We added the null hypothesis: “The null hypothesis of the study is that the development and implementation of a VLE on conscious antibiotic prescription in dentistry does not have a favorable impact on participants’ awareness, attitudes, and intention to practice.” **Line 121 (manuscript with track changes).**

METHODS:

- Line 199: the score categorization used follows a certain logic for awareness and attitudes (high level being considered only if there is total correctness) and another for intention to practice (high level for those who get around 63 to 100% of the questions right). For awareness and attitudes, getting 63% of the questions right would mean low or medium level. I would like to understand the authors' option for this categorization, and, if they deem it pertinent, I suggest putting this as a limitation of the study in the discussion.

**Response:** We added and explained the limitation: “….score categorization was a limitation as according to the percentile classification during the questionnaire validation, a high level of awareness and attitudes was achieved with 100% of correct answers whereas, for intention to practice, a high level was reached with 63% - 100% of correct answers [27]. **Line 466 (manuscript with track changes).**

RESULTS:

- Lines 250 and 257: figures 2 and 3 always compare 4 sets of data, referring to "before", "immediate", another "before" and "6 months". In my opinion, the occurrence of 2 "before" is only clear when analyzing the supporting information. Thus, it may be worth clarifying in Methods that the comparisons were made with the total sample that completed the VLE course (first "before" and "immediate" sample) and with those who answered the questionnaire after 6 months (second "before" and "6 months" sample).

**Response:** In Materials and Methods, we clarified about your suggestion: The immediate impact (“immediately after” intervention) was compared to the first measurement (“before” intervention) of the total sample of dentists that completed the course. To analyze information retention, results from dentists that answered the questionnaire at 6 months (“6 months” post-intervention) were compared to the data obtained from the same population that answered the initial questionnaire (“before” intervention). **Line 175 (manuscript with track changes).**

DISCUSSION:

- Line 438: revise English in the sentence "A study in the medical field, in which an online course was given to students and showed that >90% of the participants had a good score on activities related to basic knowledge (principles of antibiotic stewardship);".

**Response:** We modified the sentence “In a similar study, an online course given to 310 medical students showed that the majority had an average score of 90% on activities related to basic knowledge (principles of antibiotic stewardship). Moreover, 153 of these participants completed all five interactive online clinical cases, with about half of them scoring over 90% in the first attempt [36]”. **Line 441 (manuscript with track changes).**

- Line 460: the limitations paragraph is quite modest, associating the limitations mainly with COVID-19. In addition, the authors sticked a final paragraph of discussion on limitations. I suggest a distinct paragraph, inserting the following topics: 1. study design, without a control group; 2. score categorization (already indicated above in Methods, Line 199); and 3. indicate as selection bias the fact that dentists from a previous study were invited to participate in this study.

**Response:** We organized the limitation according to your suggestion. **Line 462 (manuscript with track changes).**
