# Supplementary material for: Impact of a virtual learning environment on the conscious prescription of antibiotics among Colombian dentists
Source: PLoS One. 2022 Jan 28;17(1):e0262731. doi: 10.1371/journal.pone.0262731 (PMC8797226; doi:10.1371/journal.pone.0262731)
Supplement: Attachment — Submitted filename: Responses to reviewers.docx [file pone.0262731.s004.docx]

Dear Editor:

Prof. Dr. Andrej M Kielbassa

Academic Editor

PLOS ONE

Please, in the attached file, you will find the revised manuscript PONE-D-21-04062: " Impact of a Virtual Learning Environment on the conscious prescription of antibiotics in Colombian dentists" by Angarita Diaz *et al.*, that we are submitting for publication in "PLOS ONE". We have added responses to the suggestions and comments of the reviewers and would like to thank them for all their time and consideration of our work.

Moreover, according to the comment, “I am convinced that your revised paper will be worth following, even if your revised version still would benefit from thorough re-edits and language polishing.”, *we performed the editing process on this occasion and added the certification. During the edition, one word of the manuscript's title changed: Impact of a Virtual Learning Environment on the conscious prescription of antibiotics* ***among*** *Colombian dentists"*

In relation to the comment, “Please review your reference list to ensure that it is complete and correct. If you have cited papers that have been retracted, please include the rationale for doing so in the manuscript text, or remove these references and replace them with relevant current references. Any changes to the reference list should be mentioned in the rebuttal letter that accompanies your revised manuscript. If you need to cite a retracted article, indicate the article’s retracted status in the References list and also include a citation and full reference for the retraction notice”. *We thoroughly reviewed the reference list.*

Best regards,

The authors

***RESPONSE JOURNAL REQUIREMENTS:***

**Reviewer #1:**

Dear reviewer, thanks for your comments that support us to improve the manuscript. We specify the changes and corrections made as follows:

Some concerns remained and requested minor revision.

Material and Methods

I suggest adjusting the text:

l. 128 “The present study is minimal risk research and was approved….”

for this phrase, “This study was approved….”

**Response:** We modified this phrase according to your suggestion. **Line 126. (manuscript with track changes)**

The calculus of sample size remains unclear. A total of 140 dentists were required, but how many dentists per city were needed, considering the total number of dentists per city?

**Response:** We specified the total number of required dentists per city: “At least 20 dentists per city registered at the local Health Secretary or the Sistema Integrado de Información de la Protección Social of the Ministry of Health were required.” **Line 138. (manuscript with track changes)**

Results

l. 230 “A total of 206 dentists completed the course.”

How many participants initiate the course? Please, write about the loss of the participants. It is essential to know how many participants initiated the course and how many completed the course.

**Response:** We added the number of participants that initiated the course and how many completed the course: “A total of 279 dentists initiated the VLE, of which 73.8% completed the course.” **Line 227. (manuscript with track changes).** We would like to specify that we did not ask about the reasons for course dropout in the study.

I suggest complementing the legend’s information of the Tables:

l. 237 Table 2. Sociodemographic characteristics of the participants (n = 206)

**Response:** We modified this legend according to your suggestion. **Line 235. (manuscript with track changes)**

l. 239 Table 3. Baseline levels of awareness, attitudes, and intention to practice of the participants (n = 206)

**Response:** We modified this legend according to your suggestion. **Line 237.** **(manuscript with track changes)**

The first column of Table 2 and Table 3 indicates the Absolute Number and not Frequency. The Frequency means prevalence(percentage).

**Response:** We modified this column according to your suggestion.

l. 232 “Furthermore, most dentists showed a medium level of awareness (57.3%) and attitudes (80.1%) and a high level of intention to practice (91.3%) (Table 3).” Does 80.1% represent a medium level?

**Response:** We modified the redaction as follows: “Furthermore, 57.3% and 80.1% of dentists showed a medium level of awareness and attitudes, respectively, and 91.3% had a high level of intention to practice practice (Table 3).” **Line 231.** **(manuscript with track changes).**

Why was the Wilcoxon signed-rank test used instead of the Friedman test? The comparison was made among three groups (basal, immediately after, and 6 -months post-intervention) and not only two groups.

**Response:** The comparisons were between two groups. Line 169 specified this information regarding the comparisons: “To determine the effect of the VLE on dentists, participants answered a questionnaire intended to measure their levels of awareness, attitudes, and intention to practice antibiotic prescription before, immediately after, and 6 months post-intervention. The immediate impact (“immediately after” intervention) was compared to the first measurement (“before” intervention) of the total sample of dentists who completed the course. To analyze information retention, results from dentists who answered the questionnaire at 6 months (“6 months” post-intervention) were compared to the data obtained from the same population that answered the initial questionnaire (“before” intervention).”

According to the instructions, the authors must reorder:

• Acknowledgments

• References

• Supporting information captions

**Response:** We re-ordered the sections.
